# Data from network meta-analyses can inform clinical practice guidelines and decision-making in diabetes management: perspectives of the taskforce of the guideline workshop

**DOI:** 10.1186/s12933-023-01993-3

**Published:** 2023-10-13

**Authors:** Antonio Ceriello, Helena W. Rodbard, Tadej Battelino, Frank Brosius, Francesco Cosentino, Jennifer Green, Linong Ji, Monika Kellerer, Susan Koob, Mikhail Kosiborod, Nebojsa Lalic, Nikolaus Marx, T. Prashant Nedungadi, Christopher G. Parkin, Lars Rydén, Wayne Huey-Herng Sheu, Eberhard Standl, Per Olav Vandvik, Oliver Schnell, for the Taskforce of the Guideline Workshop

**Affiliations:** 1 IRCCS MultiMedica, Via Milanese 300, 20099 Sesto San Giovanni, MI Italy; 2 Endocrine and Metabolic Consultants, 3200 Tower Oaks Blvd., Suite 250, Rockville, MD 20852 USA; 3 University Medical Center Ljubljana, and Faculty of Medicine, University of Ljubljana, Ljubljana, Slovenia; 4 University of Arizona College of Medicine, 1501 N. Campbell Ave, Tucson, AZ 85724-5022 USA; 5 Cardiology Unit, Department of Medicine, Karolinska Institute and Karolinska University Hospital, Solna, Stockholm, Sweden; 6 Duke University Medical Center, Duke Clinical Research Institute, 200 Morris St, DUMC Box 3850, Durham, NC 27715 USA; 7 Peking University People’s Hospital, 11 Xizhimen S St, Xicheng District, Beijing, China; 8 Marienhospital Stuttgart, Böheimstraße 37, 70199 Stuttgart, Germany; 9 PCNA National Office, 613 Williamson Street, Suite 200, Madison, WI 53703 USA; 10 Saint Luke’s Mid America Heart Institute and University of Missouri-Kansas City, 4401 Wornall Rd, Kansas City, MO 64111 USA; 11 The George Institute for Global Health and University of New South Wales, Sydney, Australia; 12 University Clinical Center of Serbia, University of Belgrade, Pasterova 2, 11000 Belgrade, Serbia; 13 Department of Internal Medicine I, University Hospital Aachen, RWTH Aachen University, Pauwelsstraße 30, 52074 Aachen, Germany; 14 American Heart Association, 7272 Greenville Avenue, Dallas, TX 75082 USA; 15 CGParkin Communications, Inc., 2675 Windmill Pkwy, Suite 2721, Henderson, NV 89074 USA; 16 Department of Medicine K2, Karolinska Institute, Stockholm, Sweden; 17 Institute of Molecular and Genomic Medicine, National Research Health Institutes, Zhunan, Miaoli 350 Taiwan; 18 Forschergruppe Diabetes E. V, Ingolstaedter Landstraße 1, Neuherberg, 85764 Munich, Germany; 19 Institute of Health and Society, University of Oslo, Oslo, Norway

**Keywords:** Network meta-analysis, Randomized controlled trial, Sodium glucose cotransporter 2 inhibitor, Glucose-dependent insulinotropic polypeptide, (GIP RA), Glucagon-like peptide-1 receptor agonist (GLP-1 RA), Tirzepatide, Finerenone

## Abstract

In recent years, several novel agents have become available to treat individuals with type 2 diabetes (T2D), such as sodium-glucose cotransporter-2 inhibitors (SGLT-2i), tirzepatide, which is a dual glucose-dependent insulinotropic polypeptide receptor agonist (GIP RA)/glucagon-like peptide-1 receptor agonist (GLP-1 RA), and finerenone, a non-steroidal mineralocorticoid receptor antagonist (MRA) that confers significant renal and cardiovascular benefits in individuals with (CKD). New medications have the potential to improve the lives of individuals with diabetes. However, clinicians are challenged to understand the benefits and potential risks associated with these new and emerging treatment options. In this article, we discuss how use of network meta-analyses (NMA) can fill this need.

An estimated 537 million people worldwide have diabetes, an alarming number that is projected to reach 643 million by 2030 at an annual cost of over $1 trillion (USD) [1]. Most of this cost is associated with the acute and chronic complications resulting from overall suboptimal cardiovascular risk management, including insufficient glycaemic control [2]. As reported in recent epidemiological studies, the inability to achieve optimal disease management (glycaemia, lipids, blood pressure) remains problematic for many individuals with diabetes [3–5]. However, as the rate of innovation in the development of new diabetes medications and technologies continues to accelerate, there is a growing and diverse array of treatment options that may facilitate more effective management [6, 7].

In recent years, several novel agents have become available to treat individuals with type 2 diabetes (T2D), such as sodium-glucose cotransporter-2 inhibitors (SGLT-2i). Pharmacologic innovations have also led to new first-in-class medications such as tirzepatide, which is a dual glucose-dependent insulinotropic polypeptide receptor agonist (GIP RA)/glucagon-like peptide-1 receptor agonist (GLP-1 RA) that lowers HbA1c with significant reductions in body weight [8–11]. Another new medication class is finerenone, a non-steroidal mineralocorticoid receptor antagonist (MRA) that confers significant renal and cardiovascular benefits in individuals with (CKD) [12].

 While new medications have the potential to improve the lives of individuals with diabetes, clinicians are challenged to understand the benefits and potential risks associated with these new and emerging treatment options. Traditionally, clinicians have relied on clinical practice guidelines based on evidence from cardiovascular outcome trials (CVOTs). To meet standards for trustworthy guidelines, recommendations need to be based on systematic reviews, typically meta-analyses of all available randomized controlled trials (RCTs). However, because a standard pairwise meta-analysis can only compare the efficacy or safety of two medications that have been compared in head-to-head clinical trials, it is impossible to make the same risk–benefit determination when several possible treatments are available to treat patients with the same condition. To provide effective, personalized care to their patients, clinicians need the ability to select the most appropriate treatment among several options.

The use of network meta-analyses (NMA) can fill this need. Also referred to as multiple treatment meta-analyses or mixed treatment comparisons, NMAs combine direct and indirect evidence acquired from one or more common comparators to simultaneously compare multiple treatments in a single pooled analysis [13, 14]. This approach differs from earlier neural node meta-analyses in which compounds are compared to each other for a single measure of efficacy (e.g., HbA1c) vs. current approach in which the common comparator is “standard treatment”. Direct evidence is acquired from RCTs that directly compare two medications in head-to-head assessments (e.g., intervention A vs. intervention B), while indirect evidence is acquired from RCTs assessing one or more common comparators. In the absence of a study that reports an A vs. B comparison, it is possible to make this assessment by combining studies with common comparators (e.g., A vs. C and B vs. C). Based on the direct and indirect evidence assessed, a network map is created to graphically depict the number of patients and trials assessed and the network estimate is pooled result of the direct and indirect evidence.

An example of this approach is the recent systematic review and NMA by Shi et al. [15] This NMA is an update of a previous systematic review that informed a clinical practice guideline (BMJ Rapid Recommendations), supported by the MAGIC Evidence Ecosystem Foundation. [16] In the updated NMA, investigators assessed the most current evidence of T2D medication from a larger data set of 821 trials with 471,815 patients [15]. In addition to updated evidence on SGLT-2i and GLP-RA, this NMA included studies of finerenone and tirzepatide, which are new to clinicians. Investigators grouped drug treatments by their treatment class with connections between each drug in all included trials for any outcome. This resulted in 9976 estimates of effect across 13 drugs and 11 outcomes, clearly representing an insurmountable challenge to digest for readers. To ease navigation, interpretation, and use of the evidence in decision-making, the interactive MATCH-IT tool provides user-friendly access to all comparisons and interventions (https://matchit.magicevidence.org/230125dist-diabetes/#!/) [17].

The Taskforce of the Guideline Workshop, an international multidisciplinary team including endocrinologists, cardiologists, and nephrologists, helped formulate the clinical questions and provided input into the study protocol. The aim of the Taskforce is to develop and implement a roadmap for the acceleration and harmonization of clinical guidelines and updates for diabetes, prediabetes, cardiovascular, and kidney diseases. [7, 18] (Fig. 1).

**Fig. 1:**
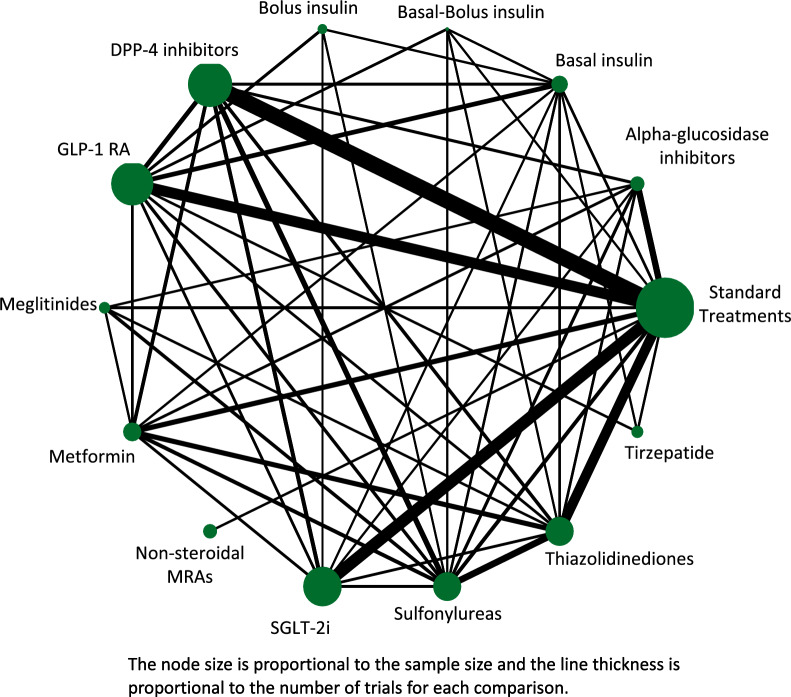
Network map for all included studies [15]

Certainty of the evidence was assessed following Grading of Recommendations Assessment, Development, and Evaluation (GRADE) guidance [19]. This approach focuses on the magnitude of the benefits, harms, and burdens of the interventions and the comparators; the quality of evidence associated with the evidence of benefits, harms, and burdens; and the underlying values and preferences of the population to whom the recommendation applies [20]. Cost, feasibility, and acceptability are also considered [21]. The GRADE approach considers only two types of evidence: randomized trials and observational studies, which are graded as high, moderate, low, and very low. A strong rating identifies recommendations in which the benefits outweigh the harms [22], whereas a *weak rating* indicates that the recommendation should be considered based on a patient’s specific needs and preferences and it should involve shared decision making [7, 23].

To categorise the relative impact of interventions, investigators defined the null effect as the decision threshold and standard treatments as the reference intervention [24, 25]. Standard treatments refer to the control/comparator group included in each study. Treatment options are displayed in rows and outcomes in columns. The cells are colour-coded to indicate the magnitude and certainty of the treatment effect in relation to the reference treatment [25].

The drugs found to be superior or inferior to standard treatments were categorised from the most effective to the most harmful, taking certainty of evidence into account. Drugs were further categorized based on the certainty of supporting evidence: “high to moderate certainty” or “low to very low certainty”. From these analyses, investigators generated a comprehensive summary of the benefits and harms of the diabetes drugs with estimates that represent the comparative effects of the drugs compared to standard treatments. To address the needs of patient groups with various comorbidities (e.g., T2D with existing CVD), the evidence summary presents the incidence of the pre-defined outcomes to be anticipated with the new treatment approaches compared with standard medical care within the five CVD/CKD risk groups. (e.g., “more” or “fewer” events per 1,000 patients) compared with standard treatments. The clinical outcomes considered in the current network meta-analysis were all-cause death, cardiovascular death, non-fatal myocardial infarction, non-fatal stroke, hospitalization for heart failure, end-stage kidney disease (ESKD), health-related quality of life (HRQoL); severe hypoglycaemia, and drug-specific adverse events. A similar summary of comparative effects was developed for individuals with T2D and CKD.

Investigators found that both the SGLT-2i and GLP-1 RA medications were effective in reducing all-cause death, cardiovascular death, non-fatal myocardial infarction, hospitalization for heart failure, and ESKD. Although only GLP-1 RAs reduced non-fatal stroke, SGLT-2i medications were shown to be superior to other medications in reducing end-stage kidney disease. For patients with T2D and CKD, it was reported that the non-steroidal MRA medication (finerenone) probably reduces hospital admissions for heart failure and end-stage kidney disease and decreases mortality. Tirzepatide appears to facilitate the largest reduction in body weight and increase in health-related quality of life (QoL) in individuals with T2D [15] followed by varying effects of the individual GLP-1 receptor agonists. The key reported harms were largely specific to each medication class; genital infections with SGLT-2 inhibitors, gastrointestinal adverse events with tirzepatide and GLP-1 receptor agonists, and hyperkalemia, leading to admission to hospital with finerenone.

RCTs remain the gold standard for direct comparison of two interventions. However, when multiple interventions or the same disease or condition are being considered, synthesis of results from RTCs of the various interventions using the NMA model ensures that all relevant direct and indirect evidence is considered. This approach generates more comprehensive and clinically useful estimates of the relative effects of multiple interventions. As demonstrated in the analysis performed by Shi et al. and the accompanying MATCH-IT tool [15, 17], the use of NMAs offers the ability to visualize and interpret a broader picture of the evidence and better understand the relative merits of each intervention when multiple interventions have been used to treat the same disease. Moreover, the NMA model may facilitate creating and updating guidelines more rapidly based on practice-changing evidence. Indeed, the recent NMA on diabetes drugs is now informing an update of the BMJ Rapid Recommendations and in Australia, both in the shape of living guidelines. The CVOT Taskforce recommends that our professional societies to consider use if this NMA to inform their guideline recommendations.

## Data Availability

Not applicable.

## Abbreviations

CKD: Chronic kidney disease
CVOT: Cardiovascular outcome trials
ESKD: End-stage kidney disease
GIP: Glucose-dependent insulinotropic polypeptide receptor agonist
GLP-1RA: Glucagon-like peptide-1 receptor agonist
GRADE: Grading of Recommendations Assessment, Development and Evaluation
MRA: Non-steroidal mineralocorticoid receptor antagonist
NMA: Network meta-analyses
QoL: Quality of life
SGLT-2i: Sodium-glucose cotransporter-inhibitors
T2D: Type 2 diabetes
USD: United States dollars
